# Psychosocial challenges among Asian adolescents and young adults with cancer: a scoping review

**DOI:** 10.1186/s12885-025-14169-x

**Published:** 2025-04-24

**Authors:** Yihui Wei, Panpan Xiao, Weishang Deng, Cho Lee Wong, Chun-Kit Ngan, Winnie Wan-Yee Tso, Alex Wing-Kwan Leung, Herbert Ho-Fung Loong, Chi Kong Li, Alexandre Chan, Yin Ting Cheung

**Affiliations:** 1 School of Pharmacy, Faculty of Medicine, The Chinese University of Hong Kong, Hong Kong SAR, China; 2 The Nethersole School of Nursing, Faculty of Medicine, The Chinese University of Hong Kong, Hong Kong SAR, China; 3 Data Science Program, Worcester Polytechnic Institute, Worcester, MA USA; 4 Department of Paediatrics & Adolescent Medicine, The University of Hong Kong, Hong Kong SAR, China; 5 Department of Paediatrics & Adolescent Medicine, Hong Kong Children’s Hospital, Hong Kong SAR, China; 6 Department of Paediatrics, Faculty of Medicine, The Chinese University of Hong Kong, Hong Kong SAR, China; 7 Department of Clinical Oncology, Faculty of Medicine, The Chinese University of Hong Kong, Hong Kong SAR, China; 8 Hong Kong Hub of Paediatric Excellence, The Chinese University of Hong Kong, Hong Kong SAR, China; 9 School of Pharmacy & Pharmaceutical Sciences, University of California, Irvine, CA USA; 10 Department of Oncology Pharmacy, National Cancer Centre Singapore, Singapore, Singapore

**Keywords:** Adolescents and young adults, Cancer, Survivorship, Work, Relationships, Infertility

## Abstract

**Background:**

Most of the landmark cohorts and reviews that assessed the psychosocial outcomes among adolescent and young adult (AYA) cancer survivors have focused on Western populations. This scoping review summarizes the existing evidence on psychosocial challenges experienced by AYAs with cancer in Asia, specifically work- and school-related outcomes, financial distress, social relationships, and concerns with infertility.

**Methods:**

A literature search was conducted on Embase and Medline for studies that (1) were published in English between 2000 and 2023, (2) recruited AYAs diagnosed with cancer between the age of 15 and 39 years, (3) were conducted in Asia, and (4) assessed outcomes related to (i) work or/and school performance, (ii) financial distress, (iii) romantic relationship or/and relationship with family and peers, and (iii) concerns with childbirth and infertility. Titles, abstracts, and full texts were screened independently by two reviewers to identify eligible studies. Information of included studies was summarized and aggregated using structured forms based on Joanna Briggs Institute’s (JBI) data extraction form. Both quantitative and qualitative studies were assessed for methodological validity using JBI Critical Appraisal Checklist.

**Results:**

Thirteen studies, enrolling a total of 1,108 survivors, reported outcomes related to work or school performance (*n* = 8), relationships with families and peers (*n* = 5), and desires or concerns regarding childbirth or infertility (*n* = 5). Although no differences in resignation rates between AYA survivors of cancer and non-cancer controls or other age groups were reported in three studies, 21%-40% of AYAs expressed concerns regarding employment or impaired work outcomes after cancer diagnosis. Studies identified health concerns and socioenvironmental factors that affected family functioning and romantic relationships. The uncertainty and the lack of information on fertility preservation were consistently reported by participants.

**Conclusions:**

Our review demonstrated differences in concerns and disparities in social support and interventions available to AYA cancer survivors among various Asian countries/regions. We found Asian studies have focused more on family relationships than peers, likely due to sociocultural nuances when compared with Western societies. Given the variability in economic development and healthcare infrastructure across Asia, region-specific healthcare policies and services are required for AYA survivors.

**Supplementary Information:**

The online version contains supplementary material available at 10.1186/s12885-025-14169-x.

## Introduction

Adolescent and young adult (AYA) patients with cancer typically comprise individuals diagnosed with cancer between the ages of 15 and 39 years [1]. Over the past decades, advancements in cancer treatment have led to a decrease in overall cancer mortality rates among AYAs [1, 2]. Given that the transition period during adolescence and young adulthood is characterized by significant developmental changes toward independence [3–6], changes in the functional outcomes of AYA survivors during their cancer trajectory are complex, including physical, mental, and psychosocial aspects [6–9]. Older adolescents face challenges during puberty and social identity development in school, whereas young adults focus on pursuing higher education, career goals, and independent social relationships [6].

In this review, we adopted the National Cancer Institute definition of “survivorship”, which focuses on the health and well-being of a person with cancer from the time of diagnosis until the end of life [10]. To enhance awareness and support for AYA survivors of cancer, the National Comprehensive Cancer Network (NCCN) has highlighted the unique psychosocial needs of this population, particularly employment and occupational opportunities, insurance/financial problems, fertility, childcare, and psychosocial support [11]. Psychosocial functioning for AYA cancer survivors involves their ability to establish relationships and fulfil their roles in various contexts, such as family, peer groups, educational or occupational settings, and the broader community. AYA cancer survivors encounter greater challenges in social functioning than the general AYA population [12], and are more likely to report psychosocial issues than older patients with cancer [13–15]. Treatment-related side effects, physical limitations, and disruptions in daily routines can also impede AYA cancer survivors’ ability to participate in social activities and maintain social connections [7]. In addition, problems related to infertility and family planning may lead to altered family dynamics in young adult survivors [9, 16–18].

Many reviews and landmark cohort studies have examined psychosocial outcomes among AYA cancer survivors [19–22]. However, most of these studies have focused on Western populations, such as US [23, 24], Italy [25], UK [26], and Canada [27]. Data on the psychosocial outcomes of AYA cancer cohorts in Asian are limited, mostly focusing on Asian women with breast cancer or older adults with cancer. The literature has suggested that psychosocial functioning outcomes may differ between Western and Asian cancer populations [28]. In addition, Asian patients diagnosed with cancer reported more unmet needs for psychosocial care and had a significantly lower quality of life than Australian [29]. These studies have indicated that racial and cultural differences may impact psychosocial outcomes of AYA cancer survivors.

This scoping review aimed to explore the existing literature on psychosocial challenges among AYA cancer survivors in Asia, specifically focusing on school or occupational performance, financial distress, romantic and family relationships, and issues related to childbirth and infertility. The findings of this review may help identify cultural nuances and unmet social needs in Asian AYA survivors of cancer, and guide the development of future psychosocial research and rehabilitation programs among the Asian population.

## Methods

### Overall approach

The protocol for this review was registered on PROSPERO in September 2023 (CRD42023459051) originally as a systematic review. However, the preliminary search showed high heterogeneity in the assessment and scope of psychosocial outcomes. The investigators decided to conduct a scoping review instead, based on the understanding that scoping reviews are better applicable to stressing the research gaps and summarizing studies with various methodologies. We followed the methodological framework recommended by Arksey and O’Malley [30] to conduct this scoping review, including: (1) identifying the research question; (2) identifying relevant studies; (3) study selection; (4) charting the data; (5) collating, summarizing and reporting the results. The Preferred Reporting Items for Systematic Review and Meta-Analysis extensions for Scoping Review (PRISMA-ScR) checklist was used to guide the review process [31].

### Literature search

A literature search was performed through two major English databases (Ovid Medline and Ovid Embase) in September 2023 to identify studies published between 2000 and 2023. The search strategy was developed based on the combination of Medical Subject Headings (MeSH) terms and keywords. A hand search was conducted by screening the reference lists of the included studies and key journals to identify publications that were potentially relevant to the research question. The specific search terms and full search strategy are presented in Supplemental Table 1.

### Eligibility criteria

While there is no consensus on the age range for AYAs across different countries, AYAs with cancer are generally defined as individuals diagnosed with cancer between the ages of 15 and 39 years [32, 33]. To include AYA cancer survivors who were diagnosed at the upper age limit (i.e., 39 years) and were recruited or evaluated for psychosocial outcomes after the completion of cancer treatment or during the cancer survivorship stage, the maximum age at recruitment/assessment for survivors included in this review was extended to 42 years. This extension of upper age limit allows us to capture the characteristics of the AYA population who were diagnosed within the specified age range and are still within the AYA category at the time of the study. Thus, this review selected studies that (1) included AYA survivors diagnosed with cancer between the age of 15 to 39 years old, who were no more than 42 years old at the time of recruitment/assessment; (2) assessed either: (i) school or work performance, (ii) financial distress, (iii) concerns related to childbirth or infertility, or (iv) intimate/romantic relationships, or relationships with children, siblings, families, and friends at any time during cancer survivorship; (3) were written in English; (4) were quantitative studies of any design (e.g., cross-sectional, longitudinal, cohort, case–control, and randomized controlled trials) or qualitative interviews or assessments; and (5) were conducted in the United Nations geoscheme for Asia.

Studies were excluded if they (1) focused on treatment effectiveness, pathophysiological analysis, or genetic polymorphisms as main outcomes; (2) reported only the proportion of related outcomes (e.g., educational attainment, marital status, or employment) as demographic characteristics and not as the main outcomes of interest; (3) were case reports, reviews or meta-analyses, comments, conference abstracts, study designs, or protocols; or (4) lacked full-text availability.

### Study selection, data extraction and data synthesis

Retrieved articles were imported into EndNote for management. Duplicates were removed both automatically and manually. Two reviewers (YW and WD) independently screened the titles and abstracts using Covidence (www.covidence.org). Furthermore, the two reviewers reviewed full texts in parallel to identify eligible articles based on the aforementioned inclusion and exclusion criteria. Disagreements were resolved through discussion with a third reviewer (YTC) until a consensus was reached.

Data extraction was independently conducted and reviewed by two investigators (YW and PX) using standardized, pilot-tested data extraction forms designed for this study based on the Joanna Briggs Institute’s (JBI) data extraction form [34]. Extracted data included (1) information on the article (title, author, year of publication, country or region, and study design); (2) characteristics of the study population (sample size, age at diagnosis, age at study, sex, cancer diagnosis, and treatment); and (3) results (specific outcome, instruments used, follow-up duration, risk factors, and control groups).

The outcomes of this review were determined a priori based on the NCCN guidelines for Adolescent and Young Adult Oncology (version 2) [11], which emphasized individual functioning (education, career, and employment), relationships (social, peer and family relationships), socioeconomic issues and infertility concerns as key psychosocial domains assessed in the AYA cancer population. Outcomes concluded from extracted data were summarized and aggregated by the corresponding domains, including (1) work or school performance, or socioeconomic outcomes, (2) romantic relationship or relationship with family and peers, and (3) concerns with childbirth and infertility.

### Quality assessment

Although quality assessment of the included studies is generally not required for a scoping review, assessment of the methodological limitations was evaluated to establish the quality of existing evidence and address variation in the study approaches. Quantitative and qualitative studies were assessed for methodological validity using JBI Critical Appraisal Checklist for Analytical Cross-Sectional Studies and JBI Critical Appraisal Checklist for Qualitative Research, respectively [35, 36]. Each article was independently appraised by two investigators (YW and PX) and any discrepancies in the ratings were resolved by discussion. In accordance with the literature [37, 38], a high methodological quality (i.e. low risk of bias) was determined if the “yes” score (suggesting that the study had addressed the possibility of bias in its design, conduct, and analysis) was 70% or higher. Studies with a “yes” score between 50 and 69% were regarded as moderate methodological quality, while those with a score of 49% or lower were rated as low methodological quality.

## Results

### Study selection and characteristics

**Fig. 1:**
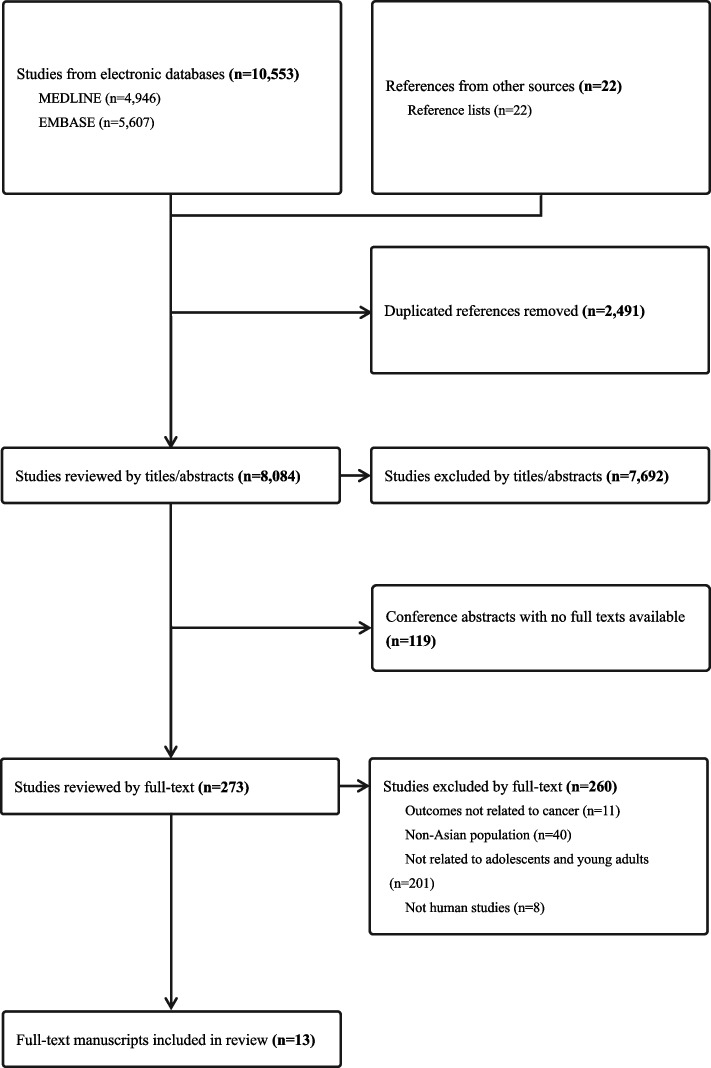
PRISMA flow chart of literature search

The flowchart of study selection and results is presented in Fig. 1. A total of 10,575 articles were extracted from the databases. Among them, there were 10,553 articles from MEDLINE (*n* = 4,946) and EMBASE (*n* = 5,607), and 22 were from other sources. After removing duplicates (*n* = 2,491), ineligible titles or abstracts (*n* = 7,692), and conference abstracts (*n* = 119), 273 full-text articles were assessed for eligibility. Finally, 13 studies that met the criteria were included for the scoping review.

Among the 13 studies (Table 1), nine were quantitative studies [39–47] and four were qualitative studies [48–51]. All the qualitative studies were considered to be of high methodological quality (Supplemental Table 2). The methodology of the quantitative studies ranged widely from low (*n* = 2) [40, 41], moderate (*n* = 2) [42, 44], to high (*n* = 5) [39, 43, 45–47] quality. Most studies (76.9%) were conducted in developed countries, including Japan (*n* = 6) [40–43, 45, 50] and Singapore (*n* = 4) [39, 46, 48, 49], whereas two studies were performed in China [47, 51] and one study was from Malaysia [44]. The total number of survivors enrolled in the 13 studies was 1,108. The majority of the studies enrolled less than 60 participants. The three studies with the largest study population were from Malaysia (*n* = 400) [44], Japan (*n* = 206) [45] and China (*n* = 150) [47]. In most studies, the proportion of the female survivors was higher than that of the male survivors, with three studies involving only female participants [42, 47, 51].

**Table 1:** Characteristics of included studies

Author	Year	Country	Sample size	Gender (% female)	Study design	Cancer diagnosis	Outcomes				
							**Work or school performance**	**Financial distress**	**Family relationships**	**Romantic relationships**	**Childbirth desires/concerns**
Chan, et al. [39]	2018	Singapore	65	44.6	Prospective longitudinal cohort	Mixed	√	√	√	√	√
Fujii, et al. [40]	2019	Japan	28	NR	Cross-sectional	Sarcoma	√				
Furui, et al. [41]	2019	Japan	36	88.9	Cross-sectional	Mixed					√
Furui, et al. [42]	2019	Japan	52	100.0	Cross-sectional	Mixed					√
Endo, et al. [43]	2020	Japan	60	85.0	Cross-sectional	Mixed	√				
Hamzah, et al. [44]	2020	Malaysia	400	56.8	Cross-sectional	Mixed	√				
Ke, et al. [48]	2020	Singapore	23	60.9	Qualitative interview	Mixed	√		√		
Tan, et al. [49]	2020	Singapore	23	60.9	Qualitative interview	Mixed	√	√			
Okamura, et al. [45]	2021	Japan	206	87.4	Cross-sectional	Mixed	√			√	
Yoshida, et al. [50]	2022	Japan	24	66.7	Qualitative interview	Mixed				√	
Qiu, et al. [51]	2023	China	12	100.0	Qualitative interview	Breast cancer			√		√
Tan, et al. [46]	2023	Singapore	29	48.3	Cross-sectional	Mixed	√				
Wu, et al. [47]	2023	China	150	100.0	Cross-sectional	Colorectal cancer					√

*NR* Not reported

Studies are arranged in chronological order

### Work or school performance

Eight studies [39, 40, 43–46, 48, 49] examined work or school performance and financial burdens (Table 2), including one prospective longitudinal study [39] and two qualitative studies [48, 49]. Two studies reported resignations from work [43, 46], but no significant differences in the resignation rate were found between AYA survivors of cancer and other age groups or healthy populations. A population-based cross-sectional survey conducted in Japan [43] indicated that 20% of participants (*n* = 750) resigned after the diagnosis of cancer; however, this proportion was not significantly higher than those in other age groups (11.4%, 12.1%, and 11.6% for the age groups of 41–49, 51–59, and > 60 years, *P* = 0.317). Another study conducted by Tan et al. in Singapore [46] revealed no significant difference in the proportion of successful employment between AYA survivors (*n* = 29) who were actively seeking jobs and their sibling controls (*n* = 23). After adjusting for age, sex, education level, and siblings’ correlation, the diagnosis of cancer was not significantly associated with decreased employment opportunities among AYA survivors compared with their healthy siblings (OR = 0.60, 95% CI: 0.07–5.07, *P* = 0.64) [46].

**Table 2:** Studies on work or school performance and financial distress

Author, year	Country	Study design	N	Control group (n)	Age at dx range in years	Age at study mean ± SD (range) in years	Follow up time	Treatment (%)	Outcomes	Assessments	Main results
**Quantitative studies**											
Chan, et al. [39], 2018	Singapore	Prospective, longitudinal cohort	65	NA	15–39	27.8 ± 6.7	‧ At diagnosis (T1) ‧ 1 month (T2) ‧ 6 months (T3)	NR	‧ Financial and insurance issues ‧ Work and school stress	The NCCN Distress Thermometer	‧ Distress about financial and insurance problems (46.2% at T1, 23.1% at T2, 23.1% at T3) ‧ Work and school issues (38.5% at T1, 18.5% at T2, 21.6% at T3)
Fujii, et al. [40], 2019	Japan	Cross-sectional	28	Childhood cancer survivors (22)	15–29	NR	Overall mean follow-up: 6 years	‧ Surgery (NR) ‧ Chemotherapy (NR) ‧ Radiation (NR)	Socio-occupational outcomes	Simplified socio-occupational disability scoring system using the PCQL- 32 and MMQL-YF	‧ Drop-out or delays in high school or college (50%, overall cohort) ‧ Unemployed or difficulties in searching for a job (56%, overall cohort) ‧ No significant difference in socio-occupational problems between AYA and childhood cancer survivors
Endo, et al. [43], 2020	Japan	Cross-sectional	60	‧ Age 40–49 years (167) ‧ Age 50–59 years (298) ‧ Age > = 60 years (225)	20–39	NR	NA	NR	Resignation after cancer diagnosis	Online structured questionnaire	‧ Resignation rate (20%) in AYA survivors; not significantly different from other age groups
Hamzah, et al. [44], 2020	Malaysia	Cross-sectional	400	NA	NR	29.1 ± 7.2 (18–40)	NA	NR	‧ Quality of working life ‧ Career engagement	‧ QWLQ-CS ‧ Career engagement scale	‧ Career engagement associated with quality of work life ‧ Effect of cancer and treatment mediated the association between quality of work life and career engagement
Okamura, et al. [45], 2021	Japan	Cross-sectional	206	NA	NR (< 39)	33.7 ± 4.3 (16–39)	NA	Chemotherapy (43.7)	‧ Change in work and school	‧ SCNS-SF34 ‧ MSPSS	‧ Decreased work/study hours (9.7%), absence from work/school (24.3%), left work/school (28.1%), changed jobs/schools (10.7%), dismissed from work (1.0%) ‧ Decreased income status (41.7%), increased income status (8.3%) ‧ Change in work/school after diagnosis associated with total supportive needs, physical/daily living needs, and psychological needs
Tan, et al. [46], 2023	Singapore	Cross-sectional	29	Healthy siblings (23)	15–39	26.6 ± 5.1	NA	‧ Chemotherapy (86.2) ‧ Surgery (48.3) ‧ Radiation (27.6) Immunotherapy (3.4)	‧ Employment status ‧ Work changes ‧ Absenteeism and presenteeism at work	‧ Structured questionnaire	‧ Fully employed (78.9%) ‧ Work reallocation (6.7%) ‧ Absenteeism within past 3 months (73.3%) ‧ Decreased work ability (40.1%) ‧ Median productivity loss due to absenteeism in the past 3 months: USD 110
**Qualitative studies**											
Ke, et al. [48], 2020	Singapore	Qualitative interview	23	NA	16–39	25 (18–39)	NA	NR	Employment support	Focus group discussions	‧ Deemed cancer history as “disadvantageous” in job search process ‧ Highlighted the need for to ensure fair hiring practices for cancer survivors, and to assist survivors in their job search
Tan, et al. [49], 2020	Singapore	Qualitative interview	23	NA	16–39		NA	‧ Chemotherapy (91.3) ‧ Radiation (30.4) ‧ Surgery (56.5)	‧ Problems with returning to work ‧ Financial issues	Focus group discussions	‧ Majority of AYA survivors expressed eagerness to return to work ‧ Highlighted challenges due to cancer complications, work restrictions, and inadequate insurance coverage

*Dx* Diagnosis, *IQR* Interquartile range, *MMQL-YF* Minneapolis-Manchester Quality of Life-Youth Form, *MSPSS* Multidimensional Scale of Perceived Social Support, *NA* Not applicable, *NCCN* National Comprehensive Cancer Network, *NR* Not reported, *PCQL- 32* Pediatric Cancer Quality of Life Inventory- 32, *QWLQ-CS* Quality of Working Life Questionnaire for Cancer Survivors, *SCNS-SF34* The short-form Supportive Care Needs Survey questionnaire, *SD* Standard deviation

Impaired outcomes at work due to the cancer experience were noted among Asian AYA survivors [40, 44–46]. Hamzah et al. [44] reported the mediating role of cancer and related treatment on the association between career engagement and quality of work among AYA cancer survivors in Malaysia (*n* = 400). Tan et al. [46] found that in Singapore, more than half of the employed survivors (*n* = 29) experienced work impairments, including reallocation of work (6.7%), absenteeism (26.7%) and decreased ability due to the lingering effects of cancer (40.0%); half of the unemployed survivors attributed their unemployment to cancer and its treatment [46]. Japanese AYA survivors with sarcoma (*n* = 28) reported poor socio-occupational quality of life scores that were comparable with those of childhood cancer survivors [40]. Furthermore, Japanese survivors who experienced changes in work or school performance after the cancer diagnosis were more likely to report unmet physical and psychological needs than those who did not experience such changes [45].

### Financial distress

A prospective longitudinal study conducted in Singapore [39] reported insurance or financial burdens experienced by AYA survivors (*n* = 65) at the time of diagnosis (46.2%) and at the 6-month follow-up (23.1%). The decrease in concerns related to insurance or finances over time was not significant (*P* = 1.000). While some of the survivors were concerned about disclosing their medical history and not being able to meet work expectations, the majority of AYA survivors (*n* = 23) from a qualitative interview in Singapore [49] expressed eagerness to return to work after cancer treatment due to financial needs. Despite the challenges, several survivors used their cancer experience to enhance job opportunities, demonstrating resilience and adaptability in the face of adversities [49]. Moreover, through qualitative studies, Ke et al. and Tan et al. emphasized the need for employment services and insurance coverage among Singaporean AYA cancer survivors [48, 49].

### Romantic relationship or relationship with families and peers

Five studies [39, 45, 48, 50, 51] reported outcomes related to relationships with peers and families, particularly focusing on partners and children (Table 3). A prospective study performed in Singapore [39] did not identify any significant changes in family-related outcomes at diagnosis and 1- and 6-month follow-ups (*n* = 65). These outcomes included issues related to childcare (9.2% vs 7.7% vs 4.6%, *P* = 0.067), family health (26.2% vs 10.8% vs 14.0%, *P* = 0.078), partner relationships (9.2% vs 7.7% vs 1.5%, *P* = 0.174), and interaction with children (9.2% vs 3.1% vs 1.5%, *P* = 0.097). However, a descriptive trend towards a decrease in self-reported problems over time was noted. Okamura et al. [45] conducted a population-based cross-sectional study in Japan (*n* = 206) and found that 35.4% of the participants were concerned regarding changes in sexual feelings.

**Table 3:** Studies on relationships with families, partners and peers

Author, year	Country	Study design	N	Control group (n)	Age at dx range in years	Age at study mean ± SD (range) in years	Follow up time	Treatment (%)	Outcomes	Assessments	Main results
**Quantitative studies**											
Chan, et al. [39], 2018	Singapore	Prospective, longitudinal cohort	65	NA	15–39	27.8 ± 6.7	‧ At diagnosis (T1) ‧ 1 month (T2) ‧ 6 months (T3)	NR	‧ Problems with childcare ‧ Dealing with partner ‧ Dealing with children	The NCCN Distress Thermometer	‧ Problems with childcare (9.2% at T1, 7.7% at T2, 4.6% at T3) ‧ Dealing with partner (9.2% at T1, 7.7% at T2, 1.5% at T3) ‧ Dealing with children (9.2% at T1, 3.1% at T2, 1.5% at T3) ‧ Concerns about family health (26.2% at T1, 10.8% at T2, 14.0% at T3)
Okamura, et al. [45], 2021	Japan	Cross-sectional	206	NA	NR (< 39)	33.7 ± 4.3 (16–39)	‧ NA	Chemotherapy (43.7)	‧ Sexuality needs	‧ SCNS-SF34 MSPSS	‧ Expressed changes in sexual feelings (35.4%)
**Qualitative studies**											
Ke, et al. [48], 2020	Singapore	Qualitative interview	23	NA	16–39	25 (18–39)	NA	NR	Family support	Focus group discussions	‧ Multiple roles of AYAs in relation to their family members ‧ Most AYA patients had children as dependents or parents as caregivers
Yoshida, et al. [50], 2022	Japan	Qualitative interview	24	NA	15–38	35.5 (24–43)	NA	NR	Romantic relationship and marriage	Semi-structured interview	Top three concerns about romantic relationship: ‧ Fertility and parenthood concerns (45.8%) ‧ Disclosure of cancer history to partners (37.5%) ‧ Recurrence, metastasis, and poor health (25.0%)
Qiu, et al. [51], 2023	China	Qualitative interview	12	NA	24–40	32.8 ± 4.1	NA	‧ Surgery (100) ‧ Chemotherapy (66.7) ‧ Radiation (58.3) ‧ Endocrine therapy (75.0) ‧ Targeted therapy (25.0) ‧ Neoadjuvant chemotherapy (25.0)	Familial and peer support	Semi-structured interview	‧ Highlighted the need for emotional support from family members and peers ‧ Expressed gratitude for the husband and maintenance of marriage bond

*Dx* Diagnosis, *IQR* Interquartile range, *MAPSS* Multidimensional Scale of Perceived Social Support, *NA* Not applicable, *NCCN* National Comprehensive Cancer Network, *NR* Not reported, *SCNS-SF34* The short-form Supportive Care Needs Survey questionnaire, *SD* Standard deviation

In a qualitative study [50], over one third of Japanese AYA survivors (*n* = 24) expressed difficulties in deciding when, how, and to what extent to disclose their cancer history to potential partners. They feared rejection due to their cancer history when establishing new romantic relationships [50]. Qiu et al. and Ke et al. highlighted the multiple roles related to family members and the lack of adequate emotional support from families [48, 51].

### Desire for or concerns regarding childbirth

Five studies [39, 41, 42, 47, 51] reported desires or concerns regarding childbirth or infertility among AYA cancer survivors (Table 4). In a longitudinal study conducted in Singapore (*n* = 65) [39], patients reported concerns regarding having children at diagnosis (18.5%) and 1-month (4.6%) and 6-month follow-ups (4.6%), but the change in these concerns over time was not significant (*P* = 0.157). Furui et al. [41] found that 25% of male survivors and 75% of female survivors had reproduction and fertility concerns, which were significantly higher than the rates among AYAs without cancer (3.0%, *P* < 0.01) (*n* = 36). Another Japanese study (*n* = 52) [42] reported that a higher proportion of AYA survivors who underwent chemotherapy expressed reproductive concerns as one of their top five “current problems” compared with AYAs without cancer (51.9% vs 3.0%, *P* < 0.01). It also reported that 21.2% of AYA survivors who initially desired having children had to abandon this desire after the cancer diagnosis [42]. Studies conducted in the metropolitan areas of China also addressed AYA survivors’ fertility concerns. Qiu et al. [51] identified a strong desire for childbearing after cancer diagnosis among Chinese AYA breast cancer survivors in Guangzhou (*n* = 150), and emphasized the crucial role of support from families, partners, society, and healthcare professionals as well as self-perception in reproductive decision-making [51]. Wu et al. [47] found that among Chinese AYA survivors with colorectal cancer in Shanghai (*n* = 12), the children’s health and their personal health were the top concerns associated with childbirth, followed by those related to pregnancy, fertility potential, and the partner’s disclosure and acceptance. They also noted that more significant reproductive concerns were observed in AYA cancer survivors with fewer children or only female children, as well as lower education level, early-stage cancer diagnosis, and poorer family functioning [47].

**Table 4:** Studies on childbirth concerns and desires

Author, year	Country	Study design	N	Control group (n)	Age at dx range in years	Age at study mean ± SD (range) in years	Follow up time	Treatment (%)	Outcomes	Assessments	Main results
**Quantitative studies**											
Chan, et al. [39], 2018	Singapore	Prospective, longitudinal cohort	65	NA	15–39	± 6.7	‧ At diagnosis (T1) ‧ 1 month (T2) ‧ 6 months (T3)	NR	Concerns about ability to have children	The NCCN Distress Thermometer	‧ Concerns about having children (18.5% at T1, 4.6% at T2, 4.6% at T3)
Furui, et al. [41], 2019	Japan	Cross-sectional	36	‧ Healthy AYAs (200) ‧ Childhood cancer survivors (76)	16–39	33.2 ± 4.8	NA	Chemotherapy (69.4)	Concerns about reproductive function and infertility	Structured questionnaire	‧ More concerns about reproductive function and infertility in AYA survivors (69.5%) than healthy controls (2.5%)
Furui, et al. [42], 2019	Japan	Cross-sectional	52	Healthy AYAs (100)	15–39	32.3 ± 6.6	NA	Chemotherapy (100)	‧ Concerns about infertility ‧ Desire for childbearing	Structured questionnaire	‧ More concerns about reproductive function in AYA survivors (51.9%) than healthy controls (3%) ‧ Diagnosed as “infertile” (19.2%) ‧ Menstrual cycle abnormalities as a late effect of therapy (36.5%) ‧ More AYA survivors reported “giving up trying for a child” (21.2%) than healthy controls (0%)
Wu, et al. [47], 2023	China	Cross-sectional	150	NA	18–40	34.7 ± 3.97	NA	‧ Surgery (100) Chemoradiotherapy (NR)	‧ Reproductive concerns	‧ RCAC Family Adaptation and Cohesion Evaluation Scale II	‧ Mean score for overall reproductive concerns: 54.78 ± 8.97 (out of 90 points) ‧ High prevalence of concerns in child's health, personal health, becoming pregnant, fertility potential, partner disclosure and acceptance
**Qualitative studies**											
Qiu, et al. [51], 2023	China	Qualitative interview	12	NA	24–40	± 4.1	NA	‧ Surgery (NR) ‧ Chemotherapy (NR) ‧ Radiation (NR) ‧ Endocrine therapy (NR) ‧ Targeted therapy (NR) ‧ Neoadjuvant chemotherapy (NR)	‧ Desire for childbearing ‧ Emotional experience during pregnancy ‧ Reproductive decision-making	Semi-structured interview	Major themes: ‧ Awareness that the cancer may impair fertility status ‧ Worries about health of unborn child affected by treatment ‧ Expressed regret if the goal of childbearing is not achieved ‧ Expressed pressure from family elders to have children ‧ Trust in doctors and science influencing reproductive decisions

*Dx* Diagnosis, *IQR* Interquartile range, *NA* Not applicable, *NCCN* National Comprehensive Cancer Network, *NR* Not reported, *RCAC* Reproductive Concerns After Cancer, *SD* Standard deviation

## Discussion

Although numerous studies have examined psychological outcomes and unmet supportive care needs in AYA cancer survivors, few have specifically focused on the downstream occupational and family outcomes in this population in Asia. To the best of our knowledge, this study is the first to summarize concerns about work/school, financial distress, relationships and concerns with infertility among AYA cancer survivors in Asian countries. Almost all the studies we reviewed were conducted in high-income or upper-middle regions/countries with excellent cancer survival rates and survivorship care. We did not identify any similar studies conducted in other developing or low- or middle- income countries (LMICs) in Asia. Our review demonstrated differences in concerns and disparities in social support and interventions available to AYA cancer survivors among various Asian countries and regions. Given that occupational achievement and family values are highly regarded in many Asian societies [52], more studies examining psychosocial functioning outcomes in AYA cancer survivors should be conducted in the future.

Similar to the Western population [53, 54], we found that 20% to 56% of the Asian AYA cancer survivors in this review expressed concerns regarding employment after their diagnosis. However, three included studies did not identify significant differences in the resignation rate between AYA cancer survivors and their counterparts (i.e. cancer survivors of other age groups, healthy populations, or sibling controls). Collectively, our review showed that Asian AYA survivors experienced pressure and distress related to employment; however, many were eager to return to work and resume a daily life similar to that of their healthy peers [19, 40, 45]. This finding highlights the importance of employment services, which include various initiatives to ensure fair hiring practices for cancer survivors.

In Western countries, there are several comprehensive programs offering extensive and integrated support to help young cancer survivors return to work. For example, the European Network of Youth Cancer Survivors (EU-CAYAS-NET) advocates for policies to reduce financial discrimination and improve access to employment for young cancer survivors, and also provides rehabilitation programs that include vocational training and psychological support [55]. Such services in Asian countries are less widespread but are emerging, especially in high-income countries. According to a survey conducted by the Economist Intelligence Unit, Japan was the representative Asian country to offer rehabilitation programs and supports for cancer survivors to return to work [56]. The Japanese government urged employers to implement supportive measures for cancer survivors, including employer policy statements, sick leave programs, and flexible working arrangements to help them balance work and treatment [57]. In addition, the Singapore Cancer Society has introduced the Return-To-Work-Programme to help cancer survivors rejoin the workforce or retaining their employment [58]. It is well documented that many European countries have established an extensive network of support programs for young cancer survivors [59, 60]. Encouragingly, Asia is also making significant strides due to the higher prioritization of cancer survivorship in public health agendas, particularly in high-income countries [61, 62]. However, such policies may differ across regions or countries in terms of the level of support provided and region-specific implementation barriers [63], and are expected to be tailored against discrimination. For example, one study in Japan reported that female cancer patients are more likely to be marginalized in the workplace than male cancer patients [64]. Key recommendations for a work reintegration program should include (1) adapting to local resource availability with dedicated budgets and infrastructure for work integration for AYA cancer survivors, (2) fostering a multidisciplinary team (including clinicians, nurses, social workers, rehabilitation specialists, and occupational therapists) to support a successful transition back to work, and (3) creating a supportive environment and policies addressing potential discrimination (such as gender or chronic disability discrimination) at the workplace.

Financial distress has been less commonly investigated and reported in Asian AYA cancer survivors than in Western populations [19–21, 23]. This may be because in contrast to the United States, most Asian countries/regions such as China, Hong Kong, Japan, Singapore, and South Korea, have a well-established universal health coverage system to cover basic cancer treatment. However, the out-of-pocket expenditure for cancer treatment may vary across countries, and as reflected by the included studies of our review, many cancer patients in Asia may still face financial burdens related to cancer treatment. A study focusing on cancer survivorship in the Indo-Pacific region highlighted that the top unmet needs of cancer survivors in this region, especially in LMICs, included access to local health services and best medical care [65]. For instance, the Korean government has established a financial support program for children and adult patients from low-income households [66, 67]. However, specialized policies and programs targeting AYA cancer survivors are limited because this demographic often receives less priority than children and older adults with cancer [68]. This lack of dedicated attention and resources for AYAs highlights a gap in the healthcare system, emphasizing the need for dedicated healthcare policies to reduce the financial burden on this age group.

Previous reviews of studies from Western countries have reported challenges in various aspects of relationships with family, partners, and peers [3, 69, 70]. Our review suggested that Asian studies have focused more on family relationships than peers. These differences might be attributed to sociocultural and socioenvironmental factors prevalent in Asian societies [71–73]. For example, Asian cultures often emphasize strong family bonds and interdependence, whereas Western AYAs might rely on a broader network of friends, peers, and support groups outside their immediate family for social connections and support [74]. Furthermore, it is considered a cultural norm for young adults in some Asian societies to live with their parents and siblings before they get married [75]. For this reason, family-oriented relationships might play a more significant role in Asian than in Western cultures [74]. Understanding and addressing these culturally specific factors can guide the development of family-targeted interventions and supportive programs for AYA cancer survivors. For example, adopting a “full-cycle, whole family, whole-person rehabilitation” approach could be crucial in survivorship care for this population [76], enabling family members to provide supportive care to patients and assist them in self-care and home responsibilities. Overall, our findings highlight the importance of contextual awareness and the need to develop and implement culturally and socially sensitive interventions in collaboration with the family members of Asian AYA cancer survivors.

Fertility rates in general have declined steadily across most developed countries, with only about 1/5 of the global livebirths recorded in southeast Asia, east Asia and Oceania [77]. However, our review still highlighted childbearing and infertility as major concerns among Asian AYA cancer survivors. The uncertainty regarding fertility status and the lack of information on fertility preservation were consistently reported by participants in the included studies. The maturity and accessibility of onco-fertility programs also varied across regions/countries in Asia. For example, Japan has established regional networks for bridging oncology and reproductive medication, and introduced a national public subsidy for fertility preservation to reduce the financial burden on cancer patients [78]. The Fertility Preservation Support Service Programme led by the Hong Kong Cancer Fund, a nongovernmental organization, provides financial assistance for fertility preservation to AYAs with cancer [79]. Cultural and societal factors also affect family planning and parenting in both Asian and Western countries. In addition, changes in traditional values and economic pressure have led to lower fertility rates in many developed countries/regions of Asia. This has caused governments to review their policies and measures to encourage childbirth. For instance, China and Japan offer up to 14 weeks of maternity leave [80], which is still shorter than that in Western countries, such as Sweden, which provides extensive parental leave, with up to 480 days per child [81]. These differences may impact reproductive decisions and highlight the need for supportive policies tailored to the context of each region. Given that more knowledge on fertility preservation options can improve psychosocial functioning [82], it is hoped that increasing numbers of Asian countries will provide AYA cancer survivors with equal access to fertility services, including financial resources, insurance coverage, and availability of fertility specialists. National and regional facilities, along with community-level support, should provide information on advancements in fertility preservation techniques and options and offer guidance on family planning, reproductive health, and assisted reproductive technologies when necessary. The ongoing support and follow-up care addressing the unique concerns of AYA patients should be integral to these efforts.

This review has several limitations that should be addressed. First, this review was limited by the small sample sizes and the narrow geographical scope of the included studies. The majority of the included studies were conducted in high-income Asian countries and single centers, which may have led to misrepresentation of the diversity of psychosocial outcomes. The paucity of literature from Central, Southern and Western Asian countries may limit the generalizability of our findings to the broader Asian context. This finding suggests that AYA oncology is still an evolving specialty in many countries of Asia. Second, it was not feasible to conduct a pooled or meta-analysis as there was much variation in outcome assessments, such as family functioning and childbearing concerns. Third, this review only included studies published in English as it is methodologically difficult to conduct search and translation processes for literature written in the multiple languages represented in Asia. Despite the limitations, this scoping review captured a wide range of studies and perspectives, offering a more holistic understanding of psychosocial outcomes where the evidence is diverse and not yet well-defined. By providing a comprehensive overview of existing research, we can identify and highlight gaps that need further investigation. Future studies should consider multi-centered approaches, collaborating with international organizations and major AYA cohorts to evaluate best practices and research priorities from Asian countries. Through knowledge sharing and collaborative research, policies and capacities can be developed to improve the overall well-being of AYA cancer survivors in Asia.

## Conclusions

This review provided an overview of the current research on psychosocial challenges among Asian AYA cancer survivors. We highlighted the unmet needs of the Asian AYA cancer population in areas such as family relationships, returning to work, and childbirth and discussed potential support mechanisms and interventions for Asian survivors. Given the variability in economic development and healthcare infrastructure across Asia, tailored policies, health insurance schemes, and healthcare services and support are required for AYA patients. Future studies should be conducted in Asian countries to comprehensively evaluate the psychosocial outcomes of Asian AYA cancer survivors. These studies could inform targeted care and interventions for long-term survivorship and boost the overall well-being of Asian AYA cancer survivors.

## Supplementary Information

Supplementary Material 1

## Data Availability

No datasets were generated or analysed during the current study.
