# Supplementary material for: Psychosocial challenges among Asian adolescents and young adults with cancer: a scoping review
Source: BMC Cancer. 2025 Apr 24;25:770. doi: 10.1186/s12885-025-14169-x (PMC12020170; doi:10.1186/s12885-025-14169-x)
Supplement: Supplementary file 1 — Supplementary Material 1 [file 12885_2025_14169_MOESM1_ESM.docx]

**Psychosocial challenges among Asian adolescents and young adults with cancer: A scoping review**

Yihui Wei, Msc^1^, Panpan Xiao, Msc^1^, Weishang Deng, Msc^1^, Jojo Cho-Lee Wong, PhD^2^, Chun-Kit Ngan, PhD^3^, Winnie Wan-Yee Tso, MBBS^4,5^, Alex Wing-Kwan Leung, MD^5,6^, Herbert Ho Fung Loong, MBBS^7^, Chi Kong Li, MBBS, MD^5,6,8^, Alexandre Chan, PharmD, MPH^9,10^, Yin Ting Cheung, PhD^1,8*^

**Affiliations:**

^1^School of Pharmacy, Faculty of Medicine, The Chinese University of Hong Kong, Hong Kong SAR, China.

^2^The Nethersole School of Nursing, Faculty of Medicine, The Chinese University of Hong Kong, Hong Kong SAR, China.

^3^Data Science Program, Worcester Polytechnic Institute, Worcester, MA, United States.

^4^Department of Paediatrics & Adolescent Medicine, The University of Hong Kong, Hong Kong SAR, China.

^5^Department of Paediatrics & Adolescent Medicine, Hong Kong Children's Hospital, Hong Kong SAR, China.

^6^Department of Paediatrics, Faculty of Medicine, The Chinese University of Hong Kong, Hong Kong SAR, China.

^7^Department of Clinical Oncology, Faculty of Medicine, The Chinese University of Hong Kong, Hong Kong SAR, China.

^8^ Hong Kong Hub of Paediatric Excellence, The Chinese University of Hong Kong, Hong Kong SAR, China.

^9^School of Pharmacy & Pharmaceutical Sciences, University of California, Irvine, CA, United States.

^10^Department of Oncology Pharmacy, National Cancer Centre Singapore, Singapore.

**Corresponding Author:** Yin Ting Cheung, PhD, Associate Professor, Email address: [yinting.cheung@cuhk.edu.hk](mailto:yinting.cheung@cuhk.edu.hk).

Supplemental Table 1. Search terms for Databases of Ovid Medline/Embase

| **Domain** |  | **Medline/Embase search terms** |
| --- | --- | --- |
| Population: Adolescents and young adults | 1 | exp Adolescent/ or exp Young Adult/ |
|  | 2 | (AYA* or adoles* or young adult* or young people or young person* or teenage* or teen* or child* or youth*).mp. |
|  | 3 | 1 or 2 |
| Survivors of all-kind cancers | 4 | (cancer* or tumo?r* or malignan* or leuk?emia* or carcinoma* or neoplasm* or sarcoma* or medulloblastoma* or lymphoma* or melanoma or oncolog*).mp. |
|  | 5 | (surviv* or patient* or population*).mp. |
| Asia | 6 | (Asia or China or Japan or Korea or Mongolia or Taiwan or Hong Kong or Brunei or Myanmar or Cambodia or Indonesia or Laos or Malaysia or Philippines or Singapore or Thailand or Timor-Leste or Vietnam).in. |
|  | 7 | 3 and 4 and 5 and 6 |
| School performance | 8 | (school* or college* or universit* or graduate* or graduation* or (educat* or course* or lesson* or class* or academ*) adj2 (performance* or attain* or level*)).mp. |
| Work performance | 9 | (employ* or occupation* or work* or career* or job* or labo?r* or vocation* or profession* or retire* or unemploy* or reemploy* or underemploy*or absenteeism* or presenteeism*).mp. |
| Socioeconomic outcomes | 10 | (financ* or income* or salary or salaries or wage* or earn* or ((health* or cancer* or disease* or person*) adj2 (economic* or insurance*))).mp. |
| Family (relationship) | 11 | (family or families or parent* or offspring* or relatives* or father* or mother* or sibling* or brother* or sister* or grandparent* or paternal or maternal).mp. |
| Romantic relationship / fertility concerns (relationship/reproduction) | 12 | (romance* or romantic relationship* or intimacy* or intimate* or sexual or infertil* or fertil* or libido* or (reproduct* adj2 (outcome* or concern* or problem* or issue* or function* or dysfunction* or disorder* or health* or satisfaction*)) or couple* or spouse* or mate* or partner* or marital or marriage* or husband* or wife or wives).mp. |
| Friend and peer (relationship) | 13 | (friend* or companion* or acquaint* or peer* or ((interpersonal or social or personal) adj2 (activit* or network*))).mp. |
|  | 14 | 8 or 9 or 10 or 11 or 12 or 13 |
|  | 15 | 7 and 14 |
| Exclude: Non-English, full text not available, published before 2000 | 16 | limit 15 to (human and english language and full text and yr="2000 -Current") |

Supplemental Table 2. Critical Appraisal for Included Studies by the Joanna Briggs Institute’s (JBI) Critical Appraisal Checklists [35-38]

| **Quantitative Studies^*^** | | | | | | | | | | | |  |
| --- | --- | --- | --- | --- | --- | --- | --- | --- | --- | --- | --- | --- |
| **Study and year** | **Q1** | **Q2** | **Q3** | **Q4** | **Q5** | **Q6** | **Q7** | **Q8** |  |  | **% Yes** | **Quality** |
| Chan, et al. [39], 2018 | √ | √ | √ | √ | × | × | √ | √ |  |  | 75.0 | High |
| Fujii, et al. [40], 2019 | × | √ | NA | U | × | × | U | √ |  |  | 25.0 | Low |
| Furui, et al. [41], 2019 | × | √ | NA | √ | NA | NA | U | √ |  |  | 37.5 | Low |
| Furui, et al. [42], 2019 | × | √ | √ | √ | NA | NA | U | √ |  |  | 50.0 | Moderate |
| Endo, et al. [43], 2020 | √ | √ | NA | √ | √ | √ | √ | √ |  |  | 87.5 | High |
| Hamzah, et al. [44], 2020 | √ | √ | NA | √ | × | × | √ | √ |  |  | 62.5 | Moderate |
| Okamura, et al. [45], 2021 | √ | √ | NA | √ | √ | √ | √ | √ |  |  | 87.5 | High |
| Tan, et al. [46], 2023 | √ | √ | √ | √ | √ | √ | √ | √ |  |  | 100 | High |
| Wu, et al. [47], 2023 | √ | √ | NA | √ | √ | √ | √ | √ |  |  | 87.5 | High |
|  |  |  |  |  |  |  |  |  |  |  |  |  |
| **Qualitative Studies^#^** | | | | | | | | | | | |  |
| **Study and year** | **Q1** | **Q2** | **Q3** | **Q4** | **Q5** | **Q6** | **Q7** | **Q8** | **Q9** | **Q10** | **% Yes** | **Quality** |
| Ke, et al. [48], 2020 | √ | √ | √ | √ | √ | × | U | × | √ | √ | 70.0 | High |
| Tan, et al. [49], 2020 | √ | √ | √ | √ | √ | × | √ | × | √ | √ | 100 | High |
| Yoshida, et al. [50], 2022 | U | √ | √ | × | √ | √ | U | √ | √ | √ | 70.0 | High |
| Qiu, et al. [51], 2023 | √ | √ | √ | √ | √ | × | √ | √ | √ | √ | 90.0 | High |

*Criteria for quantitative studies: Q1. Were the criteria for inclusion in the sample clearly defined? Q2. Were the study subjects and the setting described in detail? Q3. Was the exposure measured in a valid and reliable way? Q4. Were objective, standard criteria used for measurement of the condition? Q5. Were confounding factors identified? Q6. Were strategies to deal with confounding factors stated? Q7. Were the outcomes measured in a valid and reliable way? Q8. Was appropriate statistical analysis used?

#Criteria for qualitative studies: Q1. Is there congruity between the stated philosophical perspective and the research methodology? Q2. Is there congruity between the research methodology and the research question or objectives? Q3. Is there congruity between the research methodology and the methods used to collect data? Q4. Is there congruity between the research methodology and the representation and analysis of data? Q5. Is there congruity between the research methodology and the interpretation of results? Q6. Is there a statement locating the researcher culturally or theoretically? Q7. Is the influence of the researcher on the research, and vice- versa, addressed? Q8. Are participants, and their voices, adequately represented? Q9. Is the research ethical according to current criteria or, for recent studies, and is there evidence of ethical approval by an appropriate body? Q10. Do the conclusions drawn in the research report flow from the analysis, or interpretation, of the data?

√: yes; ×: no; U: unclear; NA: not applicable. A study with high methodological quality: % yes ≥70%; moderate methodological quality: % yes 50%-69%; low methodological quality: % yes <50%.
